# Skeletodental and soft tissue changes following treatment with herbst and pendex appliances: a retrospective CBCT study

**DOI:** 10.1007/s00784-025-06732-4

**Published:** 2026-01-12

**Authors:** Brianna Tucker, Jessica Kang, Christopher Hudson-Boyd, Sam Kadan, Bruno Saconi, Brendan T. Keenan, Richard J. Schwab, Chun-Hsi Chung, Hyeran Helen Jeon

**Affiliations:** 1 Department of Orthodontics, School of Dental Medicine, University of Pennsylvania, 240 South 40th Street, Philadelphia, PA USA; 2 School of Dental Medicine, University of Pennsylvania, Philadelphia, PA USA; 3 Private Practice, Chalfont, PA USA; 4 Division of Sleep Medicine, Department of Medicine, University of Pennsylvania Perelman School of Medicine, Philadelphia, PA USA

**Keywords:** Herbst, Pendex, Growth modification, Class II malocclusion, Cone beam computed tomography (CBCT)

## Abstract

**Objectives:**

To compare skeletodental and soft tissue changes in growing Class II patients treated with Herbst or Pendex appliances, followed by fixed edgewise appliances, using two-dimensional lateral cephalometric radiographs extracted from three-dimensional cone-beam computed tomography scans.

**Materials and methods:**

Forty-six patients were examined: 23 treated with Herbst (12.07 ± 1.49 years, 12 males/11 females) and 23 with Pendex (11.76 ± 1.18 years, 10 males/13 females). CBCT-derived lateral cephalograms were analyzed at T1 (initial), T2 (6 months post-Herbst removal or immediately post-Pendex removal), and T3 (final records after edgewise fixed appliance removal). Cephalometric analysis assessed skeletal, dental, and soft tissue changes. Repeated measures ANOVA analyzed within-group changes across the three time points, and t-tests were used to compare between-group differences at each time point and evaluate changes from T1 to T3.

**Results:**

From T1 to T3, the Herbst group exhibited a significant decrease in the SNA angle, a non-significant increase in SNB, and significant increases in mandibular dimensions, including total length, body length, corpus length, and ramus height. In contrast, the Pendex group demonstrated stable SNA values, a significant increase in SNB, and mandibular dimensional changes comparable to those observed in the Herbst group. Vertically, both groups remained stable with no significant differences in skeletal vertical parameters. Overall, no significant between-group differences were observed in skeletal, dental, or soft tissue parameters between T1 and T3.

**Conclusion/Clinical relevance:**

We did not detect statistically significant differences in overall skeletal, dental, and soft tissue changes between the two groups.

**Supplementary Information:**

The online version contains supplementary material available at 10.1007/s00784-025-06732-4.

## Introduction

**Fig. 1:**
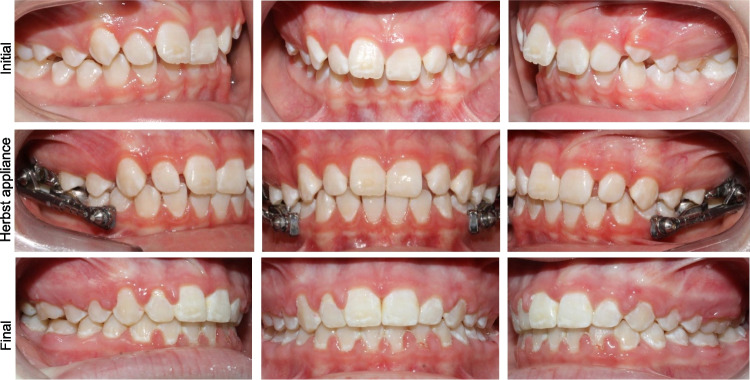
Herbst appliance. Initial, mid-treatment (during Herbst appliance), and final records

Class II malocclusion affects approximately 35% of the US population [1]. Mandibular retrognathism is the predominant etiologic factor in most skeletal Class II malocclusions [2, 3]. Fixed or removable functional appliances are common treatment options for skeletal Class II growing patients. The Herbst appliance was first introduced in the early 1900 s by Emil Herbst and regained popularity when it was reintroduced by Hans Pancherz in the 1970s, [4] becoming widely used over the past five decades (Fig. 1) [5, 6]. It positions the mandible forward during all mandibular functions to correct the Class II malocclusion. Previous studies reported maxillary growth restriction, anterior positioning of the glenoid fossa and chin advancement after the Herbst appliance treatment [7–11].

However, controversy exists about whether functional appliances can stimulate mandibular growth​. One study examined the long-term skeletodental changes after the use of different functional appliances such as the bionator, Herbst, twin block, and mandibular anterior repositioning appliance (MARA) using lateral cephalograms and compared them with untreated skeletal Class II malocclusions [12]. They observed no significant differences among the various treatments and matched control in the long term. DiBiase et al. observed that the initial increase in mandibular length following functional appliance treatment was lost over time, with treatment outcomes ultimately comparable to those of patients treated without functional appliances [13]. Recently, through 3-dimensional (3D) superimposition Souki et al. observed the significantly forward displacement of mandible, posterior and superior condylar growth and posterior ramus growth in the Herbst group compared to Class II non-orthopedic treatment control group after 8months of treatment [14]. Moreover, Taylor et al. compared the treatment outcomes between the Herbst and Pendulum group and observed a significantly greater increase in mandibular length, anterior displacements of Pogonion and the mesial displacement of the lower first molars in the Herbst group compared to the Pendulum group using 3D superimposition [7]. Therefore, the extent to which the Herbst appliance can achieve clinically meaningful long-term mandibular growth remains a topic of ongoing debate, underscoring the need for further investigation using 3D cone beam computed tomography (CBCT).

**Fig. 2:**
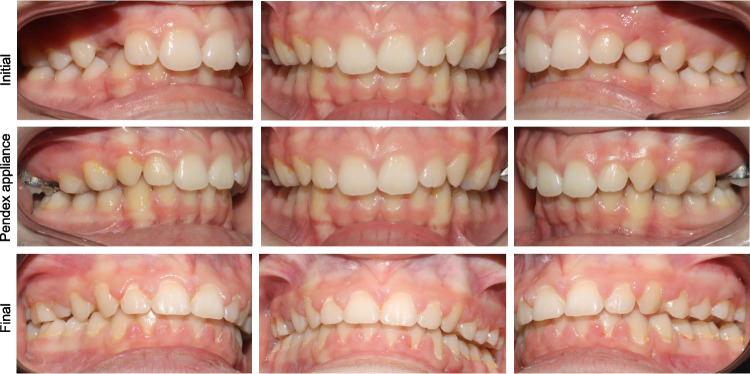
Pendex appliance. Initial, mid-treatment (during Pendex appliance), and final records

**Fig. 3:**
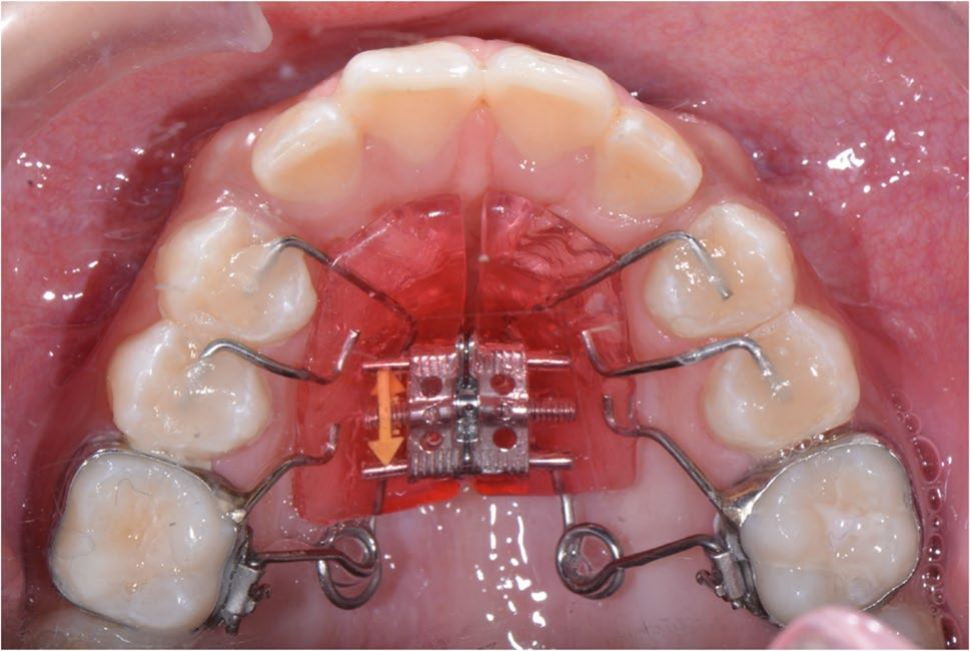
Pendex appliance (occlusal view)

For patients with Class II malocclusion who exhibit less severe skeletal discrepancies or who are past their peak growth period, maxillary molar distalization may serve as an effective treatment option. The Pendex appliance, a modified version of the Pendulum appliance incorporating a palatal expander, is particularly useful for Class II malocclusions associated with transverse discrepancies requiring expansion along with molar distalization (Fig. 2 and 3) [15]. Treatment effects of the Pendex/Pendulum appliance are primarily dental effects which correct class II malocclusions by distalization and distal tipping of the maxillary first molars, mesialization and mesial tipping of the maxillary premolars, intrusion of maxillary first molars, extrusion of maxillary second premolars, and slight proclination of maxillary incisors [16]. Studies indicate that approximately 63–76% of the space created by the Pendulum appliance results from molar distalization, while the remaining 24–37% is due to mesial movement of maxillary second premolars [16, 17]. One study reported clockwise mandibular rotation resulting from maxillary molar distalization with the Pendulum appliance [16], whereas other studies observed minimal changes in vertical dimensions [18, 19]. Overall, the primary effects of the Pendex appliance are dentoalveolar rather than skeletal.

To date, most studies on craniofacial growth have relied on two-dimensional (2D) lateral cephalometric radiographs, which have inherent limitations affecting image quality and landmark identification. These limitations include variability in patient head positioning, superimposition of bilateral craniofacial structures, and radiographic magnification. In contrast, 3D CBCT images can be reformatted to produce orthogonal 2D lateral cephalograms, effectively addressing these limitations [20–22]. Orthogonal CBCT-derived lateral cephalograms provide more accurate cranial measurements compared to conventional cephalometric radiographs [20, 22].

This study aims to evaluate the treatment outcomes of the Herbst appliance followed by fixed orthodontic appliances in growing patients with skeletal Class II malocclusion and to compare these outcomes with those of patients treated using the Pendex appliance. Specifically, we will assess positional changes of the maxilla and mandible, differences in mandibular growth, dental changes involving the maxillary and mandibular incisors and first molars, and the resulting profile changes.

## Materials and methods

### Study population

This retrospective study was approved by the Institutional Review Board at the University of Pennsylvania (#851012). This study examined the treatment effects of Herbst and the Pendex appliances, followed by fixed appliances. The study comprised 46 patients from one private practice (Chalfont, PA) with 23 treated with the Herbst appliance (12.07 ± 1.49 years, 12 males/11 females) and 23 treated with the Pendex appliance (11.76 ± 1.18 years, 10 males/13 females). The Pendex group served as the non-growth modification control, since their treatment did not intentionally alter the maxillomandibular sagittal relationship. The inclusion criteria were: (i) unilateral or bilateral Class II molar relationship of at least half-cusp width, (ii) minimal or no mandibular arch crowding, and (iii) non-extraction treatment plans. Patients with a history of orthodontic treatment, permanent tooth extractions, or craniofacial surgeries were excluded. Following the initial orthodontic records, Herbst group patients received a stainless-steel crown Herbst appliance on the maxillary first molars, combined with a maxillary expander and a mandibular lingual holding arch on the first molars. For all patients in this study, patients and their parents were instructed to carry out one turn per day (0.25 mm/turn) for 30–35 days. Following expansion, the telescopic arms were placed, and the Herbst appliance was activated until the patient achieved an anterior edge-to-edge bite, which was maintained for at least one year. For the Pendex group, the Pendex appliance was delivered with bands on the maxillary first molars and occlusal rests on the premolars or primary molars. The maxillary expansion was completed with 30–35 turns. After distalization of the maxillary first molars was complete, a Nance holding arch was placed for retention. Both groups subsequently underwent fixed edgewise orthodontic appliance treatment. Generally, the Herbst appliance is primarily indicated for skeletal Class II cases with a retrusive mandible to advance the lower jaw during growth. In contrast, the Pendex appliance is mainly indicated for dental Class II and transverse problems, serving to distalize the upper molars and expand a narrow maxillary arch rather than alter jaw position.

### CBCT analysis

**Fig. 4:**
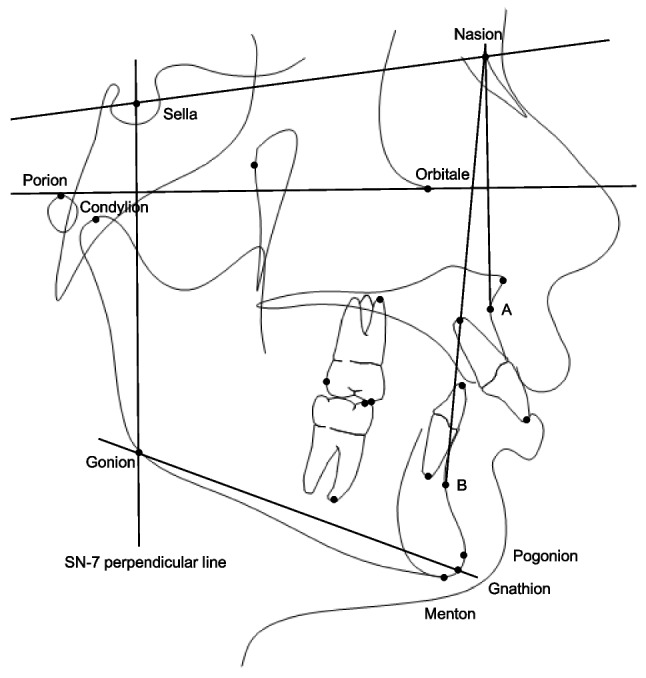
Cephalometric landmarks and measurements

CBCTs were taken using i-CAT™ machine (KaVo Imaging, Hatfield, PA, USA) at three time points, T1 (initial), T2 (6 months after the Herbst appliance removal; immediate after removal of Pendex appliance) and T3 (final records following braces removal). In the Herbst group, T2 CBCT scans were obtained within six months following the removal of the Herbst appliance to allow for partial relapse and occlusal settling, as approximately 90% of occlusal relapse occurs during the first six months post-treatment [23]. In the Pendex group, T2 CBCT scans were taken immediately after appliance removal, followed by the placement of a Nance holding arch until the initiation of fixed edgewise treatment. Using Dolphin Imaging 3D software (version 12.0; Dolphin Imaging & Management Solutions, Chatsworth, CA, USA), CBCT images were oriented in frontal and lateral views. In the frontal view, the inferior orbital rims were positioned symmetrically and parallel to the floor, and the midsagittal line was established through the nasion, anterior nasal spine, and midpoint of the chin. In the lateral view, the Frankfort horizontal line was oriented parallel to the floor. CBCT scans were further adjusted to closely align the orbital rims and posterior borders of the mandibular rami with corresponding structures on the contralateral side. After proper orientation, orthogonal lateral cephalograms (with 0% magnification) were generated, viewing the right side of the face toward the midpoint of the left maxillary central incisor, following previously described methods [22]. These lateral cephalograms were traced, and measurements were recorded for each time point as detailed in Table 1 and Fig. 4.

**Table 1:** Cephalometric measurements and definitions

Cephalometric measure	Definitions
*Skeletal Sagittal*	
SNA (º)	The angle between 3 point landmarks S, N and A points
Midfacial Length (mm)	The distance between Condylion and A point
SNB (º)	The angle between 3 point landmarks S, N and B points
Mandibular Length (mm)	The distance from Condylion to Gnathion
Mandibular Body Length (mm)	The distance from Gonion to Menton
Corpus Length (mm)	The distance from Gonion to Pogonion
ANB (º)	The angle formed by the intersection of N-A and N-B lines
Wits (mm)	The perpendicular distance between points A and B on the occlusal plane
Angle of Convexity (º)	The angle formed between Nasion, A point and Pogonion
Mx/Md Difference (mm)	The difference between mandibular length (Co-Gn) and maxillary length (Co-A)
*Skeletal Vertical*	
SN-GoGn (º)	The angle between SN and Go-Gn
FMA (MP-FH) (º)	The angle formed by the intersection of the Frankfort horizontal plane and the mandibular plane
Mandibular Plane Angle (º)	The angle formed by the intersection of the Frankfort horizontal plane and Go-Gn
Gonial Angle (º)	The angle formed between Articulare, Constructed Gonion and Constructed Gnathion
Ramus Height (mm)	The distance from Condylion to Gonion
*Dental Sagittal*	
U1-SN (º)	The angle formed by the long axis of the maxillary incisor to the SN plane
U1-NA (º)	The angle formed by the long axis of the maxillary incisor to a line from N to A
U1-NA (mm)	The distance between the tip of the maxillary incisor and a line from N to A
IMPA (º)	The angle measured through the long axis of the mandibular incisor in relation to the Gonion-Menton line
L1-NB (º)	The angle formed by the long axis of the mandibular incisor to a line from N to B
L1-NB (mm)	The distance between the tip of the mandibular incisor and a line from N to B
Interincisal angle (º)	The angle measured between the axes of the most labial maxillary and mandibular incisors
Overjet (mm)	The horizontal distance from the maxillary incisor tip to the mandibular incisor tip
U6 AP Position (mm)	The horizontal distance from the mesiobuccal cusp tip of the maxillary first molar to the SN-7 perpendicular line passing through S
L6 AP Position (mm)	The horizontal distance from the mesiobuccal cusp tip of the mandibular first molar to the SN-7 perpendicular line passing through S
U6 Angle (º)	The angle measured through the long axis of the mesiobuccal cusp to the mesiobuccal root apex of the maxillary first molar in relation to the SN line
L6 Angle (º)	The angle measured through the long axis of the mesiobuccal cusp to the mesial root apex of the mandibular first molar in relation to the mandibular plane angle (Menton-Gonion)
U6-PTV (mm)	The distance between the most distal point of the distal contour of the upper molar and Pt Vertical
*Dental Vertical*	
Overbite (mm)	The vertical distance from the maxillary incisor tip to the mandibular incisor tip
U6 Vertical Position (mm)	The vertical distance from the SN-7 line to the mesiobuccal cusp of the maxillary first molar
L6 Vertical Position (mm)	The vertical distance from the SN-7 line to the mesiobuccal cusp of the mandibular first molar
*Soft Tissue*	
Upper Lip to E-line (mm)	The upper lip position in relation to E-line (from the tip of the nose [Pronasale] to soft tissue Pogonion)
Lower Lip to E-line (mm)	The lower lip position in relation to E-line
Angle of Facial Convexity (º)	The angle formed from soft tissue Glabella (g'), subnasale (sn’) and Pogonion (Pog)

### Statistics

Descriptive statistics were used to summarize participant characteristics, with means and standard deviations reported for continuous variables, and frequencies and percentages for categorical ones. Change scores were calculated as absolute differences between baseline and later timepoints (T2 and T3) to quantify longitudinal changes within each group. Baseline differences between groups were evaluated using two-tailed unpaired *t*-tests or Mann–Whitney *U* tests for continuous variables, and chi-square tests for categorical data. Assumptions for parametric tests were checked by visually inspecting histograms, examining the Shapiro–Wilk test for normality, and testing variance homogeneity. Intraexaminer reliability was assessed by having the same examiner (B.T.) re-measure ten subjects at least four weeks after the initial measurements.

Changes over time within the Herbst and Pendex groups were analyzed using repeated-measures ANOVA or the Friedman test, depending on data distribution. When overall (omnibus) tests were significant, post-hoc pairwise comparisons followed: linear mixed-effects models were used after repeated-measures ANOVA and Wilcoxon signed-rank tests after the Friedman test. Multiple comparisons of cephalometric outcomes were corrected using the Hochberg step-up method, with significance determined by the adjusted alpha level to maintain a family-wise error rate of 5%. To compare treatment effects between Herbst and Pendex at the final timepoint (T3) and, in complementary analyses, for the change from T1 to T3, additional regression models were fit. These models were adjusted for both baseline (T1) values of the outcome variable and follow-up duration (e.g., T3 minus T1 in months). This approach helped isolate treatment effects while accounting for pre-treatment differences and variability in treatment exposure time. Effect sizes were computed to assess the magnitude of differences: Cohen’s d for parametric data (small: |0.2|, medium: |0.5|, large: |0.8|) and Cliff’s Delta (δ) for non-parametric data (small: |0.15|, medium: |0.33|, large: |0.47|). Analyses were conducted using Stata/SE version 16.1 (StataCorp, College Station, TX, USA).

## Results

The intraexaminer error between two measurement sessions was assessed using the intraclass correlation coefficient (ICC). 10 Herbst samples and 10 Pendex samples were randomly selected and re-traced by the examiner. The ICC for the Herbst group ranged from 0.78–0.99, indicating good to excellent intraexaminer reliability. The ICC for the Pendex group ranged from 0.91–0.99, indicating excellent reliability (Supplementary Table 1).

### Treatment outcomes of the Herbst appliance group

The average treatment periods of the Herbst group were 28.35 ± 7.92 (T1-T2), 10.78 ± 8.19 (T2-T3) and 39.48 ± 7.86 months (T1-T3) (Table 2). Successful Class II correction was achieved in the Herbst group from T1-T2, supported by significant reductions in ANB, Wits and angle of convexity values, based on Hochberg-adjusted comparisons (Table 3). From T1-T2, SNA significantly decreased from 82.60 ± 3.61° to 81.64 ± 3.29°. Significant increases were observed in mandibular length, body length, and corpus length, although SNB did not change significantly from T1-T2. Vertical skeletal parameters, such as SN-GoGn, FMA, and mandibular plane angle, remained stable. From T2 to T3, skeletal relationships remained largely stable, with no significant Class II correction or changes in maxillary and mandibular measurements or skeletal vertical patterns. From T1-T3, we observed significant reductions in ANB, Wits and angle of convexity values, based on Hochberg-adjusted comparisons (Table 3). From T1-T3, SNA significantly decreased from 82.60 ± 3.61° to 81.29 ± 2.98°. Although significant increases were observed in mandibular length, body length, corpus length, and ramus height, the changes in SNB from T1-T3 were not statistically significant (*p* = 0.08). Skeletal vertical patterns remained stable from T1 to T3, as indicated by non-significant changes in SN-GoGn, FMA, and the mandibular plane angle.

**Table 2:** Demographics

Characteristics		Herbst group (*n* = 23)	Pendex group (*n* = 23)	*p*-value
Sex		12 M, 11 F	10 M, 13 F	0.5550 ^‡^
Age at T1 (years)		12.07 ± 1.49	11.76 ± 1.18	0.2399 ^†^
Treatment Periods (months)	T2-T1	28.35 ± 7.92	13.69 ± 7.16	< 0.0001 *^†^
	T3-T2	10.78 ± 8.19	30.78 ± 12.29	< 0.0001 *^†^
	T3-T1	39.48 ± 7.86	45 ± 10.11	0.0446

^*^ = Statistically significant at Hochberg’s adjusted α-level. ^**†**^ = indicates a Mann–Whitney-based *p*-value, ^**‡**^ = indicates a Pearson Chi-Square-based *p*-value; all other *p*-values are derived from independent samples t-test

**Table 3:** Herbst treatment outcomes

Cephalometric measure	T1 (*n* = 23)		T2 (*n* = 23)		T3 (*n* = 23)		*p*-value	Significant pairwise comparisons**
	Mean	SD	Mean	SD	Mean	SD		
*Skeletal Sagittal*								
SNA (º)	82.60	3.61	81.64	3.29	81.29	2.98	0.0022*	T3-T1, T2-T1
Midfacial Length (mm)	83.50	5.19	85.37	5.02	86.07	5.12	0.0003*	T3-T1, T2-T1
SNB (º)	77.04	3.73	77.81	3.95	77.80	3.88	0.0865	NS
Mandibular Length (mm)	102.73	7.35	107.82	7.71	109.79	7.42	< 0.001*	T3-T1, T3-T2, T2-T1
Mandibular Body Length (mm)	62.44	5.11	65.30	5.06	66.48	4.36	< 0.001*	T3-T1, T2-T1
Corpus Length (mm)	66.46	5.25	69.15	5.33	70.08	4.59	< 0.001*	T3-T1, T2-T1
ANB (º)	5.37	2.29	4.02	2.04	3.61	2.23	< 0.001*	T3-T1, T2-T1
Wits (mm)	3.18	2.94	0.20	2.63	0.27	2.35	< 0.001*	T3-T1, T2-T1
Angle of Convexity (º)	10.22	5.44	7.30	5.40	6.29	5.89	< 0.001*	T3-T1, T2-T1
Mx/Md Difference (mm)	19.24	3.62	22.44	4.81	23.71	3.82	< 0.001*	T3-T1, T3-T2, T2-T1
*Skeletal Vertical*								
SN-GoGn (º)	30.69	5.22	31.41	5.97	30.99	6.28	0.3906	NS
FMA (MP-FH) (º)	24.49	5.02	24.45	5.69	23.74	6.00	0.1247	NS
Mandibular Plane Angle (º)	18.03	4.70	18.21	5.25	17.46	5.60	0.1899	NS
Gonial Angle (º)	120.44	6.13	121.37	6.09	119.92	6.06	0.0225	T3-T2
Ramus Height (mm)	50.80	4.49	54.02	5.45	55.78	4.65	< 0.001*	T3-T1, T3-T2, T2-T1
*Dental Sagittal*								
U1-SN (º)	107.42	9.55	105.21	4.44	108.17	6.92	0.1855 ^†^	NS
U1-NA (º)	24.81	9.27	23.55	4.36	26.87	6.07	0.1128	NS
U1-NA (mm)	5.14	2.86	3.97	1.78	5.00	2.36	0.0225	NS
IMPA (º)	97.83	6.55	98.11	6.94	96.59	6.83	0.4319	NS
L1-NB (º)	28.10	5.75	29.53	7.28	27.74	6.54	0.2209	NS
L1-NB (mm)	5.56	2.72	7.00	3.36	6.81	3.15	< 0.0001*	T3-T1, T2-T1
Interincisal Angle (º)	121.73	9.88	122.90	9.37	121.80	8.32	0.7856	NS
Overjet (mm)	6.71	1.91	2.50	1.09	3.33	0.89	< 0.0001* ^†^	T3-T1, T3-T2, T2-T1
U6 AP Position (mm)	38.20	6.05	39.21	6.40	40.47	6.16	< 0.0027*	T3-T1
L6 AP Position (mm)	36.94	6.53	40.63	6.33	41.17	6.14	< 0.0001*	T3-T1, T2-T1
U6 Angle (º)	73.76	4.56	74.40	5.47	75.61	4.85	0.1478	NS
L6 Angle (º)	85.56	3.66	82.33	4.74	81.44	4.74	< 0.0001*	T3-T1, T2-T1
U6-PTV (mm)	15.77	3.90	17.44	3.97	19.28	4.04	< 0.0001*	T3-T1, T2-T1, T3-T2
*Dental Vertical*								
Overbite (mm)	3.11	1.63	1.19	1.12	1.54	0.98	0.0005* ^†^	T3-T1, T2-T1
U6 Vertical Position (mm)	−59.57	4.93	−62.76	4.58	−64.00	4.66	< 0.0001*	T3-T1, T3-T2
L6 Vertical Position (mm)	−60.13	5.13	−63.93	4.62	−65.03	4.71	< 0.0001*	T3-T1, T2-T1
*Soft Tissue*								
Upper Lip to E-line (mm)	−0.68	2.56	−3.20	2.82	−3.10	2.57	< 0.0001*	T3-T1, T2-T1
Lower Lip to E-line (mm)	0.87	2.93	0.27	3.44	0.25	3.25	0.1364	NS
Angle of Facial Convexity (g'-sn'-Pog) (º)	149.33	4.98	151.10	6.24	150.66	5.97	0.0024*	T3-T1, T2-T1

^**^ Pairwise comparisons performed if repeated-measures ANOVA or Friedman *p* < 0.05; * = Statistically significant at Hochberg’s adjusted α-level; ^†^ = indicates a Friedman-based *p*-value; all other values are derived from repeated-measures ANOVA

Dentally, from T1-T2 upper incisors were unchanged. There were significant changes to the lower incisors sagittal position from T1-T2. L1-NB (mm) significantly increased from 5.56 ± 2.72 to 7.00 ± 3.36 mm, while the inclination indicated by L1-NB (°) and IMPA (°) remained stable. In addition, upper first molars demonstrated insignificant difference while lower first molars showed significant mesial movement. There was a significant decrease in overjet (6.71 ± 1.91 to 2.50 ± 1.09 mm) and overbite (3.11 ± 1.63 to 1.19 ± 1.12 mm). Between T2 and T3, overjet significantly increased to 3.33 ± 0.89 mm at T3 while overbite remained stable. From T1-T3, notably, the inclination of the lower incisors remained stable, as indicated by non-significant changes in IMPA and L1-NB (°). However, L1-NB (mm) significantly increased from 5.56 ± 2.72 to 6.81 ± 3.15 mm. Both the maxillary and mandibular first molars exhibited significant mesial movement. From T1 to T3, the maxillary first molars moved mesially by 2.55 mm, while the mandibular first molars mesialized by 4.24 mm. In addition, overjet decreased significantly from 6.71 ± 1.91 mm at T1 to 3.33 ± 0.89 mm at T3 and overbite decreased significantly from 3.11 ± 1.63 mm to 1.54 ± 0.98 mm.

Significant soft tissue changes were observed from T1 to T2, with the upper lip to E-plane distance change from –0.68 ± 2.56 mm to –3.20 ± 2.82 mm. This measurement remained stable from T2 to T3. In contrast, the position of the lower lip showed no significant change throughout the observation period. The angle of facial convexity increased significantly from T1 to T2 (149.33 ± 4.98° to 151.10 ± 6.24°) and remained stable between T2 and T3. The upper lip to E-line measurement significantly changed from −0.68 ± 2.56 mm at T1 to −3.10 ± 2.57 mm at T3, with the most substantial changes occurring between T1 and T2. In contrast, changes in the lower lip to E-line measurement were not statistically significant. Additionally, the angle of facial convexity increased significantly from T1 to T2.

### Treatment outcomes of the Pendex appliance group

The average treatment periods of the Pendex group were 13.69 ± 7.16 (T1-T2), 30.78 ± 12.29 (T2-T3) and 45 ± 10.11 months (T1-T3) (Table 2). In contrast to the Herbst group, SNA remained stable across all time intervals (T1–T2, T2–T3, and T1–T3) (Table 4). ANB showed a significant decrease from T2 to T3. Significant increases in mandibular skeletal measurements, including mandibular length, body length, and corpus length, were observed from both T1 to T2 and T2 to T3. Additionally, SNB increased significantly from T2 to T3. Vertically, measurements such as Sn-GoGn, FMA, and the mandibular plane angle remained stable throughout both time intervals (T1–T2 and T2–T3). From T1 to T3, SNA remained stable from 82.40 ± 3.46 to 82.44 ± 3.27° while SNB demonstrated a significant increase from 78.38 ± 2.89 to 79.44 ± 2.84°. In addition, mandibular length, mandibular body length, corpus length and ramus height significantly increased from T1 to T3, similar to the Herbst group. Due to the mandibular growth from T1 to T3, ANB and angle of convexity significantly decreased from 4.00 ± 1.48° to 3.02 ± 1.88° and from 7.40 ± 3.82° to 4.29 ± 4.89°, respectively. From T1 to T3, SN-GoGn showed a significant decrease, changing from 30.99 ± 3.51° to 29.83 ± 3.48°, and mandibular plane angle also decreased from 17.77 ± 2.79° to 16.50 ± 2.42°.

**Table 4:** Pendex treatment outcomes

Cephalometric measure	T1 (*n* = 23)		T2 (*n* = 23)		T3 (*n* = 23)		*p*-value	Significant pairwise comparisons**
	Mean	SD	Mean	SD	Mean	SD		
*Skeletal Sagittal*								
SNA (º)	82.40	3.46	82.70	3.69	82.44	3.27	0.5858	NS
Midfacial Length (mm)	82.41	3.87	84.57	3.32	85.28	3.90	< 0.0001*	T3-T1, T2-T1
SNB (º)	78.38	2.89	78.58	2.90	79.44	2.84	0.0033*	T3-T1, T3-T2
Mandibular Length (mm)	103.51	3.73	106.51	4.79	110.10	5.16	< 0.0001*	T3-T1, T3-T2, T2-T1
Mandibular Body Length (mm)	64.44	3.32	65.60	3.55	68.77	2.76	< 0.0001*	T3-T1, T3-T2, T2-T1
Corpus Length (mm)	68.35	3.15	69.61	3.24	72.39	2.76	< 0.0001*	T3-T1, T3-T2, T2-T1
ANB (º)	4.00	1.48	4.15	1.62	3.02	1.88	< 0.0001*	T3-T1, T3-T2
Wits (mm)	0.20	2.21	0.49	2.69	0.02	2.51	0.4163	NS
Angle of Convexity (º)	7.40	3.82	7.23	4.08	4.29	4.89	< 0.0001*	T3-T1, T3-T2
Mx/Md Difference (mm)	21.12	3.08	21.94	3.53	24.83	3.12	< 0.0001*	T3-T1, T3-T2
*Skeletal Vertical*								
SN-GoGn (º)	30.99	3.51	30.67	3.96	29.83	3.48	0.0274	T3-T1
FMA (MP-FH) (º)	24.04	3.11	23.73	2.94	22.93	2.56	0.0481	NS
Mandibular Plane Angle (º)	17.77	2.79	17.47	2.85	16.50	2.42	0.0103*	T3-T1
Gonial Angle (º)	119.04	6.26	119.85	6.28	119.40	7.00	0.2950	NS
Ramus Height	49.69	3.39	51.72	3.84	53.90	3.98	< 0.0001*	T3-T1
*Dental Sagittal*								
U1-SN (º)	100.28	7.56	100.23	8.14	108.19	5.36	< 0.0001*	T3-T1, T3-T2
U1-NA (º)	17.89	5.78	17.52	6.53	25.72	6.08	< 0.0001*	T3-T1, T3-T2
U1-NA (mm)	2.58	2.12	2.75	1.88	4.40	2.27	< 0.0001*	T3-T1, T3-T2
IMPA (º)	92.35	5.82	93.63	7.30	94.81	6.09	0.2244	NS
L1-NB (º)	24.16	5.98	25.33	7.36	26.70	4.99	0.1428	NS
L1-NB (mm)	4.52	2.65	4.66	2.80	5.50	2.34	0.0039*	T3-T1, T3-T2
Interincisal Angle (º)	133.95	10.71	133.00	12.50	124.56	8.63	< 0.0001*	T3-T1, T3-T2
Overjet (mm)	3.54	1.02	3.88	1.02	3.05	1.15	0.0146*	T3-T2
U6 AP Position (mm)	37.60	4.51	34.00	5.25	40.92	4.65	< 0.0001*	T3-T1, T3-T2, T2-T1
L6 AP Position (mm)	37.69	4.34	37.93	4.21	41.43	4.57	< 0.0001*	T3-T1, T3-T2
U6 Angle (º)	73.98	4.96	60.08	10.99	78.06	4.12	< 0.0001*	T3-T1, T3-T2, T2-T1
L6 Angle (º)	84.23	4.40	80.63	7.07	78.86	8.06	0.0004* ^†^	T3-T1, T2-T1
U6-PTV (mm)	15.16	3.30	14.21	3.09	19.90	3.44	< 0.0001*	T3-T1, T2-T1
*Dental Vertical*								
Overbite (mm)	3.20	1.66	2.86	1.88	1.10	0.78	< 0.0001*	T3-T1, T3-T2
U6 Vertical Position (mm)	−59.31	3.64	−61.89	5.01	−64.15	3.39	< 0.0001*	T3-T1, T3-T2, T2-T1
L6 Vertical Position (mm)	−58.63	3.52	−60.21	4.81	−63.11	3.50	< 0.0001*	T3-T1, T3-T2, T2-T1
*Soft Tissue*								
Upper Lip to E-line (mm)	−2.30	2.34	−2.27	2.29	−4.22	2.55	< 0.0001*	T3-T1, T3-T2
Lower Lip to E-line (mm)	−0.63	2.63	−0.73	2.85	−1.50	3.10	0.0360*	NS
Angle of Facial Convexity (g'-sn'-Pog) (º)	152.43	3.51	151.5	3.75	153.28	4.12	0.0019*	T3-T2

^**^ Pairwise comparisons performed if repeated-measures ANOVA or Friedman *p* < 0.05; * = Statistically significant at Hochberg’s adjusted α-level; ^†^ = indicates a Friedman-based *p*-value; all other values are derived from repeated-measures ANOVA

Dentally, from T1 to T2 the inclination and sagittal positions of maxillary and mandibular incisors were stable. Although, from T2-T3 significant upper incisor proclination occurred with U1–SN increasing from 100.23 ± 8.14 to 108.19 ± 5.36° and lower incisors also protruding (L1–NB from 4.66 ± 2.80 to 5.50 ± 2.34 mm). Overjet and overbite did not significantly change from T1-T2 although from T2-T3 both significantly decreased. Upper first molars were distalized from T1-T2 but from T2-T3 both upper and lower first molars exhibited significant mesial movement from T2–T3. From T1-T3 the anteroposterior position and inclination of upper incisors increased significantly. The inclination of lower incisors remained relatively stable from T1-T3, while the bodily position (L1-NB) increased significantly from 4.52 ± 2.65 mm at T1 to 5.50 ± 2.34 mm at T3. Overall, from T1-T3, both the maxillary and mandibular first molars mesialized. Additionally, the angulation of the maxillary first molars exhibited significant distal tipping, decreasing from 73.98 ± 4.96° to 60.08 ± 10.99°. Similar to the Herbst group, the overbite decreased from 3.20 ± 1.66 to 1.10 ± 0.78 mm. Overjet increased slightly from T1 to T2 during Pendex appliance therapy but subsequently decreased from T2 to T3.

From T1 to T2, no significant soft tissue changes were observed. However, from T2 to T3, the upper lip retracted significantly, and the angle of facial convexity increased significantly. Over the entire observation period from T1 to T3, the upper lip to E-line distance showed a significant decrease, primarily due to the retraction of the upper anterior teeth. Additionally, the angle of facial convexity increased significantly between T2 and T3.

### Comparison between Herbst and Pendex appliance groups

At T1, among skeletal measurement values only Wits appraisal was significantly larger in the Herbst group compared to the Pendex group (Table 5). In the dental domain, the Herbst group showed significantly greater proclination and protrusion of the upper incisors, with higher values for U1 – NA (°), U1 – NA (mm), IMPA and overjet compared to the Pendex group. By T3, however, final cephalometric values were largely similar between the groups across skeletal, dental, and soft tissue measurements with no statistically significant differences (Table 6). We included comparisons at T2 and from T1 to T2 between the Herbst and Pendex groups in Supplemental Tables 2 and 3. However, due to the significant differences in treatment timing between T1 and T2, our primary focus was on the overall treatment changes observed from T1 to T3.

**Table 5:** Comparison of herbst and pendex at T1

Cephalometric measure	Herbst (*n* = 23)		Pendex (*n* = 23)		Effect size	*p*-value
	Mean	SD	Mean	SD		
*Skeletal sagittal*						
SNA (º)	82.60	3.61	82.40	3.46	0.06	0.8489
Midfacial Length (mm)	83.50	5.19	82.41	3.87	0.24	0.4249
SNB	77.04	3.73	78.38	2.89	-0.40	0.1919
Mandibular Length (mm)	102.73	7.35	103.51	3.73	-0.13	0.6512
Mandibular Body Length (mm)	62.44	5.11	64.44	3.32	-0.46	0.1218
Corpus Length (mm)	66.46	5.25	68.35	3.15	-0.44	0.1458
ANB	5.37	2.29	4.00	1.48	0.38	0.0295 ^†^
Wits (mm)	3.18	2.94	0.20	2.21	1.14	0.0003 *
Angle of Convexity	10.22	5.44	7.40	3.82	0.60	0.0479
Mx/Md Difference (mm)	19.24	3.62	21.12	3.08	-0.56	0.0645
*Skeletal Vertical*						
SN-GoGn	30.69	5.22	30.99	3.51	-0.07	0.8201
FMA (MP-FH)	24.49	5.02	24.04	3.11	0.11	0.7205
Mandibular Plane Angle (º)	18.03	4.70	17.77	2.79	0.06	0.8259
Gonial Angle	120.44	6.13	119.04	6.26	0.22	0.4491
Ramus Height (mm)	50.80	4.49	49.69	3.39	0.28	0.3499
*Dental Sagittal*						
U1-SN	107.42	9.55	100.28	7.56	0.83	0.0073
U1-NA	24.81	9.27	17.89	5.78	0.90	0.0040 *
U1-NA (mm)	5.14	2.86	2.58	2.12	1.01	0.0013 *
IMPA	97.83	6.55	92.35	5.82	0.88	0.0045 *
L1-NB	28.10	5.75	24.16	5.98	0.67	0.0276
L1-NB (mm)	5.56	2.72	4.52	2.65	0.39	0.1963
Interincisal Angle	121.73	9.88	133.95	10.71	-1.19	0.0002 *
Overjet (mm)	6.71	1.91	3.54	1.02	2.07	< 0.0001 *
U6 AP Position (mm)	38.20	6.05	37.60	4.51	0.11	0.7027
L6 AP Position (mm)	36.94	6.53	37.69	4.34	-0.13	0.6478
U6 Angle	73.76	4.56	73.98	4.96	-0.05	0.8753
L6 Angle	85.56	3.66	84.23	4.40	0.33	0.2709
U6-PTV (mm)	15.77	3.90	15.16	3.30	0.17	0.5676
*Dental Vertical*						
Overbite (mm)	3.11	1.63	3.20	1.66	-0.07	0.6761^†^
U6 Vertical Position (mm)	-59.57	4.93	-58.63	3.52	-0.22	0.4592
L6 Vertical Position (mm)	-60.13	5.13	-59.31	3.64	-0.18	0.5361
*Soft Tissue*						
Upper Lip to E-line (mm)	-0.68	2.56	-2.30	2.34	0.66	0.0308
Lower Lip to E-line (mm)	0.87	2.93	-0.63	2.63	0.54	0.0756
Angle of Facial Convexity (g'-sn'-Pog)	149.33	4.98	152.43	3.51	-0.72	0.0188

^*^ = Statistically significant at Hochberg’s adjusted α-level; ^†^ = indicates a Mann–Whitney-based *p*-value; all other values are derived from independent samples t-test. Effect size: Cohen’s d for t-test (small: 0.2, medium: 0.5, large: 0.8) and Cliff’s delta for the Mann–Whitney test (small: 0.15, medium: 0.33, large: 0.47). Effect size calculated as: Herbst – Pendex

**Table 6:** Comparison of herbst and pendex at T3, adjusted for baseline (T1) values of the modeled variable and follow-up duration between T1 and T3

Cephalometric measure	Herbst (*n* = 23)		Pendex (*n* = 23)		Effect size	*p*-value
	Mean	95% CI	Mean	95% CI		
*Skeletal Sagittal*						
SNA	81.18	80.43, 81.93	82.55	81.80, 83.30	−0.79	0.0143
Midfacial Length (mm)	85.77	84.28, 87.26	85.58	84.09, 87.07	0.06	0.8592
SNB (º)	78.43	77.54, 79.32	78.80	77.92, 79.69	−0.19	0.5707
Mandibular Length (mm)	110.29	108.13, 112.45	109.60	107.44, 111.76	0.14	0.6631
Mandibular Body Length (mm)	67.14	65.92, 68.36	68.11	66.89, 69.34	−0.35	0.2790
Corpus Length (mm)	70.74	69.59, 71.88	71.74	70.59, 72.89	−0.38	0.2361
ANB	3.02	2.41, 3.63	3.61	3.00, 4.22	−0.42	0.1978
Wits (mm)	−0.48	−1.28, 0.32	0.77	−0.03, 1.58	−0.73	0.0460
Angle of Convexity	4.93	3.45, 6.41	5.64	4.16, 7.13	−0.21	0.5119
Mx/Md Difference (mm)	24.44	23.19, 25.69	24.11	22.86, 25.36	0.12	0.7251
*Skeletal Vertical*						
SN-GoGn	31.01	29.77, 32.25	29.81	28.57, 31.05	0.42	0.1857
FMA (MP-FH)	23.46	22.36, 24.56	23.20	22.10, 24.30	0.10	0.7417
Mandibular Plane Angle	17.18	16.09, 18.27	16.78	15.69, 17.87	0.16	0.6075
Gonial Angle (º)	118.86	117.70, 120.02	120.45	119.30, 121.61	−0.60	0.0623
Ramus Height (mm)	55.64	54.04, 57.23	54.05	52.45, 55.64	0.43	0.1713
*Dental Sagittal*						
U1-SN	107.38	104.83, 109.92	108.99	106.44, 111.53	−0.28	0.3965
U1-NA	26.58	24.01, 29.15	26.01	23.44, 28.58	0.10	0.7696
U1-NA (mm)	4.54	3.58, 5.50	4.86	3.90, 5.82	−0.15	0.6601
IMPA	95.71	92.91, 98.51	95.69	92.88, 98.49	−0.00	0.9922
L1-NB	26.78	24.45, 29.10	27.66	25.34, 29.98	−0.17	0.6045
L1-NB (mm)	6.39	5.67, 7.11	5.92	5.20, 6.64	0.28	0.3682
Interincisal Angle	123.26	119.48, 127.04	123.10	119.33, 126.88	0.02	0.9569
Overjet (mm)	3.32	2.76, 3.88	3.07	2.51, 3.63	0.25	0.5877
U6 AP Position (mm)	40.45	38.77, 42.14	40.94	39.25, 42.62	−0.12	0.6916
L6 AP Position (mm)	41.56	39.81, 43.30	41.04	39.29, 42.78	0.13	0.6831
U6 Angle	75.97	74.23, 77.72	77.69	75.95, 79.44	−0.43	0.1773
L6 Angle	81.03	78.84, 83.22	79.27	77.08, 81.45	0.35	0.2692
U6-PTV (mm)	19.32	18.21, 20.43	19.86	18.75, 20.97	−0.21	0.5043
*Dental Vertical*						
Overbite (mm)	1.50	1.13, 1.88	1.13	0.76, 1.51	0.43	0.1759
U6 Vertical Position (mm)	−63.87	−65.22, −62.51	−63.24	−64.59, −61.88	−0.20	0.5216
L6 Vertical Position (mm)	−64.96	−66.32, −63.59	−64.23	−65.59, −62.87	−0.23	0.4597
*Soft Tissue*						
Upper Lip to E-line (mm)	−3.83	−4.62, −3.05	−3.49	−4.27, −2.70	−0.20	0.5527
Lower Lip to E-line (mm)	−0.52	−1.39, 0.36	−0.73	−1.60, 0.14	0.11	0.7375
Angle of Facial Convexity (g'-sn'-Pog)	152.05	150.82, 153.28	151.88	150.66, 153.11	0.06	0.8540

No *p*-values were statistically significant at Hochberg’s adjusted α-level. All *p*-values reflect group differences from covariate-adjusted linear regression models. Effect size calculated as SMD = adjusted group difference ÷ model residual SD (RMSE); similar to Cohen’s d but based on regression-adjusted estimates (small: 0.2, medium: 0.5, large: 0.8). Effect size calculated as: Herbst – Pendex

From T1 to T3, after adjusting for baseline (T1) values and differences in follow-up duration, the Herbst group exhibited a greater decrease in SNA compared to the Pendex group (−1.33°, 95% CI: −2.07 to −0.58 vs. 0.05°, 95% CI: −0.70 to 0.80), with an effect size of 0.79, approaching the threshold for a large effect. However, this difference did not remain statistically significant after correction for multiple comparisons (Table 7). Similarly, the Herbst group showed a greater reduction in Wits appraisal (− 2.17 mm, 95% CI: − 2.97 to − 1.37) compared to the Pendex group (− 0.92 mm, 95% CI: − 1.72 to − 0.12), with a corresponding effect size of 0.73. Although not statistically significant, this represents a potentially meaningful skeletal sagittal improvement. Changes in mandibular dimensions, including mandibular length, mandibular body length, corpus length, and ramus height, showed small to moderate effect sizes (range: 0.14 to 0.43) but did not reach statistical significance. No significant between-group differences were observed in dental outcomes, including U1 and L1 inclination or sagittal position, and effect sizes were uniformly small (< 0.2). Similarly, soft tissue changes, such as upper lip to E-line, lower lip to E-line and angle of facial convexity, did not differ significantly between groups and were associated with small effect sizes.

**Table 7:** Comparison of herbst and pendex treatment effects from T1 to T3, adjusted for baseline (T1) values of the modeled variable and follow-up duration between T1 and T3

Cephalometric measure	Herbst (*n* = 23)		Pendex (*n* = 23)		Effect size	*p*-value
	Mean	95% CI	Mean	95% CI		
*Skeletal Sagittal*						
SNA	−1.33	−2.07, −0.58	0.05	−0.70, 0.80	−0.79	0.0143
Midfacial Length (mm)	2.82	1.33, 4.31	2.63	1.13, 4.12	0.06	0.8592
SNB	0.72	−0.17, 1.61	1.09	0.21, 1.98	−0.19	0.5707
Mandibular Length (mm)	7.16	5.00, 9.33	6.48	4.32, 8.64	0.14	0.6631
Mandibular Body Length (mm)	3.70	2.47, 4.92	4.67	3.45, 5.89	−0.35	0.2790
Corpus Length (mm)	3.33	2.19, 4.48	4.34	3.19, 5.48	−0.38	0.2361
ANB	−1.66	−2.27, −1.05	−1.08	−1.69, −0.47	−0.42	0.1978
Wits (mm)	−2.17	−2.97, −1.37	−0.92	−1.72, −0.12	−0.73	0.0460
Angle of Convexity	−3.88	−5.36, −2.40	−3.17	−4.65, −1.69	−0.21	0.5119
Mx/Md Difference (mm)	4.26	3.01, 5.51	3.93	2.68, 5.18	0.12	0.7251
*Skeletal Vertical*						
SN-GoGn	0.17	−1.07, 1.41	−1.03	−2.27, 0.21	0.42	0.1857
FMA (MP-FH)	−0.80	−1.90, 0.30	−1.06	−2.16, 0.04	0.10	0.7417
Mandibular Plane Angle	−0.72	−1.81, 0.37	−1.12	−2.21, −0.03	0.16	0.6075
Gonial Angle	−0.88	−2.04, 0.27	0.71	−0.44, 1.87	−0.60	0.0623
Ramus Height (mm)	5.40	3.80, 6.99	3.81	2.21, 5.40	0.43	0.1713
*Dental Sagittal*						
U1-SN	3.53	0.98, 6.08	5.14	2.59, 7.69	−0.28	0.3965
U1-NA (º)	5.23	2.66, 7.80	4.67	2.10, 7.23	0.10	0.7696
U1-NA (mm)	0.68	−0.28, 1.64	1.00	0.04, 1.96	−0.15	0.6601
IMPA	0.62	−2.18, 3.42	0.60	−2.20, 3.40	−0.00	0.9922
L1-NB	0.65	−1.67, 2.97	1.53	−0.79, 3.86	−0.17	0.6045
L1-NB (mm)	1.36	0.64, 2.08	0.89	0.17, 1.61	0.28	0.3682
Interincisal Angle	−4.58	−8.36, −0.80	−4.73	−8.51, −0.96	0.02	0.9569
Overjet (mm)	−1.81	−2.36, −1.25	−2.06	−2.62, −1.50	0.25	0.5877
U6 AP Position (mm)	2.55	0.87, 4.23	3.03	1.35, 4.72	−0.12	0.6916
L6 AP Position (mm)	4.24	2.49, 5.99	3.72	1.97, 5.47	0.13	0.6831
U6 Angle	2.10	0.36, 3.84	3.82	2.08, 5.57	−0.43	0.1773
L6 Angle (º)	−3.86	−6.05, −1.67	−5.62	−7.81, −3.44	0.35	0.2692
U6-PTV (mm)	3.85	2.74, 4.96	4.39	3.28, 5.50	−0.21	0.5043
*Dental Vertical*						
Overbite (mm)	−1.65	−2.03, −1.28	−2.02	−2.40, −1.65	0.43	0.1759
U6 Vertical Position (mm)	−4.76	−6.12, −3.41	−4.14	−5.49, −2.78	−0.20	0.5216
L6 Vertical Position (mm)	−5.24	−6.60, −3.88	−4.51	−5.87, −3.15	−0.23	0.4597
*Soft Tissue*						
Upper Lip to E-line (mm)	−2.34	−3.13, −1.56	−2.00	−2.78, −1.21	−0.20	0.5527
Lower Lip to E- line (mm)	−0.64	−1.51, 0.23	−0.85	−1.72, 0.02	0.11	0.7375
Angle of Facial Convexity (g'-sn'-Pog) (º)	1.17	−0.06, 2.40	1.00	−0.22, 2.23	0.06	0.8540

No *p*-values were statistically significant at Hochberg’s adjusted α-level. All *p*-values reflect group differences from covariate-adjusted linear regression models. Effect size calculated as SMD = adjusted group difference ÷ model residual SD (RMSE); similar to Cohen’s d but based on regression-adjusted estimates (small: 0.2, medium: 0.5, large: 0.8). Effect size calculated as: (T3 − T1 change in Herbst) − (T3 − T1 change in Pendex)

## Discussion

Still, the current literature regarding the effects of Class II functional appliances remains inconclusive. While some studies report mandibular growth stimulation, others show no significant differences compared to untreated controls [24–26]. In this study, we evaluated the skeletal, dental, and soft tissue changes associated with the Herbst appliance compared to the Pendex appliance, which served as a non–growth modification control. To ensure accurate landmark identification and avoid anatomical overlap, we utilized lateral cephalograms extracted from 3D CBCT scans rather than conventional radiographs. Finally, we compared the overall treatment outcomes from T1 to T3 between the Herbst and Pendex groups, both of which subsequently underwent fixed edgewise appliance therapy, reflecting standard clinical practice.

The Herbst appliance has been widely used to treat moderate skeletal Class II malocclusion in growing children, restricting maxillary growth similarly to headgear and distalizing the maxillary molars [27]. Consistent with previous studies [12, 27–30], we observed the “headgear effect,” characterized by a significant decrease in SNA (−1.33°) in the Herbst group from T1-T2. However, a more recent CBCT-based study by Farouk et al. evaluating the treatment effects of the Herbst appliance reported no significant changes in the SNA angle [31]. This discrepancy in findings may be attributed to the shorter treatment duration in their study, which followed patients for an average of 7 months, compared to our longer observation period (T1–T2: 28.35 ± 7.92 months). Moreover, this headgear effect was not observed in our Pendex group. From T1 to T3, there was no significant difference in mandibular size increase between the Herbst and Pendex groups, supporting the concept that the Herbst appliance does not substantially promote mandibular growth beyond normal growth potential. Similar increases in mandibular length, mandibular body length, corpus length, ramus height, and SNB angle were observed in both the Herbst and Pendex groups, despite the distinct mechanisms employed by these two appliances. Conversely, Taylor et al. demonstrated a significant increase in mandibular length (CoGn) observed in the Herbst group (7.3 ± 3.5 mm) compared to the Pendulum group (4.6 ± 4.5 mm) after an average treatment period of 2.8 ± 0.8 years and 2.5 ± 0.7 years respectively, using a 3D CBCT superimposition [7]. Irezli and Baysal observed significantly greater increases in SNB angle and effective mandibular length (Co-Gn) in a Herbst group compared to a maxillary molar distalization group after approximately one year of treatment [32]. These discrepancies could be attributed to differences in analysis methods and treatment durations between studies. In our study, although the reductions in ANB angle and Wits appraisal from T1 to T3 were larger in the Herbst group compared to the Pendex group, they were not statistically significant. Nevertheless, these trends align with the findings reported in previous Herbst appliance studies [12, 24, 32–34].

Vertically, there were no statistically significant differences in FMA, SN-GoGn and mandibular plane angle between the Herbst and Pendex groups from T1-T3. Consistent with our findings, Irezli and Baysal reported insignificant changes in SN-GoGn in the Herbst group (0.02 ± 2.78°) and a maxillary molar distalization group (1.06 ± 2.31°) after over 1 year treatment [32]. Burkhardt et al. compared the treatment outcomes of the Herbst and Pendulum appliances after 28–29.5 months (Herbst) and 31.6 months (Pendulum) using lateral cephalograms [34]. While the mandibular plane angle slightly decreased after Herbst appliance treatment (−0.4 ± 1.8° for Acrylic Herbst and −0.3 ± 1.4° for crown Herbst) and pogonion were slightly anteriorly located, the slight downward and backward rotation of the mandible occurred in the pendulum patients with an increase in mandibular plane angle (1.2 ± 2.3°) [34]. Another study examining Distal Jet and Pendulum appliances reported only a minimal increase, less than 1 degree, in the mandibular plane angle following distalization and subsequent fixed appliance treatment [35]. Chung and Wong reported that untreated skeletal Class II subjects aged 9–18, categorized into low-, average-, and high-angle groups, generally showed mandibular forward rotation (decreased MP-SN), with the rotation more pronounced in the low-angle group compared to the high-angle group [36].

In addition to skeletal changes, dental changes following Herbst appliance treatment also contributed to Class II malocclusion correction and were similar to those observed with the Pendex appliance. Similar to our findings, Taylor et al. observed an insignificant difference in the small U1 proclination of 0.3 ± 8.7° (Herbst) and 0.8 ± 9.7° (Pendulum) and anteroposterior displacement of 0.1 ± 2.6 mm (Herbst) and 0.5 ± 2.7 mm (Pendulum) using 3D superimposition [7]. However, existing literature shows conflicting results. Burkhardt et al. observed a greater increase in maxillary incisor angulation in the Pendulum group compared to both crown and acrylic Herbst groups [34]. Other studies using 2D cephalograms have reported similar ranges of U1 proclination for the Herbst and Pendulum appliances [27, 34, 37]. Interestingly, we found that the changes in IMPA and L1—NB (° and mm) from T1-T3 were insignificant between Herbst and Pendex groups, while previous studies demonstrated the IMPA increased significantly during the Herbst appliance treatment [6, 32, 38–40]. To mitigate mandibular incisor proclination, some clinicians use miniscrews in conjunction with the Herbst appliance. Manni et al. reported only 1.6° of mandibular incisor proclination when miniscrews were combined with the Herbst appliance, compared to 3.7° with the Herbst appliance alone [41]. Similar outcomes were found in a meta-analysis by Dboush et al., indicating an average reduction of 5.49° in mandibular incisor proclination when miniscrews were utilized with the Herbst appliance [42]. Additionally, interproximal reduction of the lower incisors can help maintain mandibular incisor inclination. Notably, L1-NB (mm) increased from T1 to T3 in both groups. However, these changes were not statistically significant when compared between groups.

The changes in the horizontal, vertical and angular position of the maxillary and mandibular first molars were similar between the Herbst and Pendex groups from T1-T3. Maxillary first molars mesialized by 2.55 mm in the Herbst group and 3.03 mm in the Pendex group (an average treatment duration of 39.48 ± 7.86 months for Herbst and 45 ± 10.11 years for Pendex). Taylor et al. observed 0.6 ± 1.7 mm of distal movement of maxillary first molars using the Pendulum and 1.4 ± 2.1 mm of mesial movement using the Herbst appliances when compared before and immediately after removal of the edgewise appliances (an average treatment duration of 2.8 ± 0.8 years for Herbst and 2.5 ± 0.7 years for Pendulum) [7]. The mandibular first molars mesialized similarly in the Herbst group and the Pendex group from T1-T3. Burkhardt et al. also observed mesial movement of the mandibular molars in the Herbst appliance and the pendulum appliance groups, followed by fixed appliances [34]. Vertically, the maxillary and mandibular first molar position changes were similar between the Herbst and Pendex groups, consistent with previous studies [34, 43]. In the Herbst group from T1 to T2, despite observing a headgear effect (SNA decreased from 82.60 ± 3.61° at T1 to 81.64 ± 3.29° at T2), the upper first molar AP position changed from 38.20 ± 6.05 mm at T1 to 39.21 ± 6.40 mm at T2. Possible explanations for the mesial movement of the maxillary first molars include: (i) relapse occurring within the 6 months after removal of the Herbst appliance; (ii) mesial movement into space created by expansion or leeway space; and (iii) natural mesial migration due to growth (approximately 0.6 mm per year) [44].

Soft tissue changes, including upper and lower lip positions to E-line and angle of facial convexity, were similar between the Herbst and Pendex appliances from T1-T3. The upper lips became more retrusive at the end of both treatment compared to the initial records, similar to previous studies [34, 45]. We suggest that lip retraction relative to the E-line resulted from maxillary incisor retraction and natural nasal growth. The overall increase of facial convexity from T1- T3 in both groups were statistically insignificant. Similarly, Baysal and Uysal compared the effects of Twin Block and Herbst appliances on soft tissue profile and found upper lip retrusion and an increase in soft tissue convexity at the end of both treatments [46].

There are several studies to compare the treatment outcomes among different anchorage system and anchorage forms of Herbst appliances. Pancherz and Hansen compared five different Herbst appliance anchorage designs, including premolar, premolar–molar, Pelott, labial–lingual, and Class III elastics, and reported that none effectively prevented anterior movement of the mandibular incisors and molars [47]. They observed that roughly 80% of incisor movement and 20% of molar movement relapsed following treatment. Weschler and Pancherz assessed the efficiency of the banded and cast splint anchorage forms of Herbst appliances and reported that none could prevent an anchorage loss [48]. Hägg et al. compared splinted and banded Herbst appliances and reported no significant differences in dentofacial treatment effects between the two designs [49].

During treatment with functional appliances, adaptive bone remodeling occurs within the condylar and glenoid fossa regions. Initially, this newly formed bone comprises predominantly Type III collagen, which exhibits limited mechanical strength and stability. Subsequently, the bone matrix undergoes progressive remodeling, characterized by the replacement of Type III collagen with the structurally superior and mechanically robust Type I collagen. Premature removal of functional appliance can result in the resorption of immature bone, influenced by the rebound effects from adjacent musculature and soft tissues. Therefore, a treatment duration of approximately 12 months is recommended to allow adequate bone maturation and ensure stable, long-term orthopedic correction [50, 51].

While the inclusion of an untreated control group would have strengthened the study, such data are unavailable as no open-access CBCT datasets exist for untreated Class II patients. Additionally, withholding orthodontic intervention in adolescents with skeletal Class II malocclusion for research purposes would present ethical challenges. To address these constraints while maintaining comparability in sex, age, and vertical growth pattern, a cohort of Class II patients treated with molar distalization was selected as a practical “pseudo-control.” This group was appropriate because their treatment protocol did not intentionally modify the maxillomandibular sagittal relationship.

3D CBCT minimizes superimposition and magnification errors, enhances landmark visualization, and enables true orthogonal imaging, allowing accurate assessment of craniofacial asymmetry, airway morphology, and skeletal remodeling. It also permits reconstruction of standardized 2D cephalograms for traditional analysis, improving diagnostic precision and reproducibility. Currently, no standardized protocol exists for 3D cephalometric measurements or superimposition, with methods varying by reference structure and software (e.g., ITK-SNAP, 3D Slicer). Taylor et al. employed 3D CBCT superimpositions to assess the effects of Herbst and Pendulum appliances by quantifying landmark displacements across the x, y, and z axes [7]. While such methods capture spatial changes, they do not directly correspond to conventional cephalometric parameters (e.g., SNA, SNB, ANB) that remain clinically interpretable and widely used. Therefore, we elected to extract 2D lateral cephalograms from the 3D CBCT data to perform standardized cephalometric analyses. This approach aligns with the American Board of Orthodontics’ recommended superimposition method using cranial base landmarks and represents the established standard for evaluating skeletal and dental changes across time points.

Our study has several limitations. First, the T1–T2 duration differed substantially between the Herbst and Pendex groups due to their inherently distinct treatment protocols. Although statistical adjustments were made (Supplemental Tables 2 and 3), the average T1–T2 interval in the Herbst group was nearly twice that of the Pendex group, precluding direct comparison of isolated appliance effects. Consequently, our analysis focused on overall treatment changes from T1 to T3. Moreover, the use of Class II elastics between T2 and T3, in combination with differing treatment durations and sample characteristics, may have influenced the dental outcomes in both groups. Second, both groups underwent maxillary expansion, which could have affected the treatment results. Third, the absence of an untreated Class II control group limits our ability to separate normal growth changes from appliance-induced effects in the Herbst group. Fourth, the sample size was relatively modest and no a priori power analysis was conducted. Based on the achieved sample size (*n* = 46; 23 per group), we estimate that the study had 80% power to detect a large between-group effect size (Cohen’s *d* ≥ 0.84) at a significance level of 0.05. This suggests that the study may have been underpowered to detect small or moderate effects. Therefore, non-significant findings should be interpreted with caution, as they may reflect limited statistical power rather than a true absence of treatment effect. Future studies with larger samples are needed to more definitively evaluate small to moderate treatment effects. Lastly, skeletal maturity assessment was limited due to incomplete visualization of cervical vertebrae on some CBCT scans. Using the cervical vertebral maturation (CVM) method, three Herbst patients were classified as CVM stages 1–2 and nine as stages 3–4, while eleven could not be classified. In the Pendex group, nine patients were at stages 1–2, seven at stages 3–4, and one at stages 5–6, with six unclassified due to incomplete vertebral visualization.

## Conclusions

We examined the skeletal, dental and soft tissue changes after the Herbst and Pendex appliance treatments using the lateral cephalograms extracted from CBCTs. From T1-T3 comparison, we made the following conclusions:

1. Skeletal changes: No statistically significant differences were found between the Herbst and Pendex groups in sagittal parameters (SNA, ANB, Wits appraisal) or in mandibular dimensions (total length, body length, corpus length, and ramus height). Similarly, vertical parameters (FMA, SN-GoGn, mandibular plane angle) did not differ significantly between groups.
2. Dental changes: Sagittal and vertical positional changes of the incisors and first molars were not significantly different between the two treatment modalities.
3. Soft tissue changes: Both the Herbst and Pendex groups showed comparable improvements in soft tissue profile.
4. Overall, no significant skeletal, dental, or soft tissue differences were found between the Herbst and Pendex groups. The clinical improvements observed in the Herbst group may be attributed to additional mechanisms, such as glenoid fossa remodeling in addition to headgear effects.

## Supplementary Information

Below is the link to the electronic supplementary material.

Supplementary file1 (DOCX 38 KB)

## Data Availability

Data supporting this study are available from the corresponding author upon reasonable request.
