# Supplementary material for: Skeletodental and soft tissue changes following treatment with herbst and pendex appliances: a retrospective CBCT study
Source: Clin Oral Investig. 2026 Jan 12;30(1):48. doi: 10.1007/s00784-025-06732-4 (PMC12791065; doi:10.1007/s00784-025-06732-4)
Supplement: Supplementary file 1 — Supplementary file1 (DOCX 38 KB) [file 784_2025_6732_MOESM1_ESM.docx]

**Supplementary Table 1.** ICC values

| **Cephalometric Measure** | **Herbst** | **Pendex** |
| --- | --- | --- |
| **Skeletal Sagittal** |  |  |
| SNA (°) | 0.905 | 0.992 |
| Midfacial Length (mm) | 0.97 | 0.984 |
| SNB (°) | 0.965 | 0.985 |
| Mandibular Length (mm) | 0.988 | 0.985 |
| Mandibular Body Length (mm) | 0.967 | 0.96 |
| Corpus Length (mm) | 0.97 | 0.981 |
| ANB (°) | 0.968 | 0.983 |
| Wits (mm) | 0.966 | 0.965 |
| Angle of Convexity (°) | 0.983 | 0.992 |
| Mx/Md Difference (mm) | 0.978 | 0.989 |
| **Skeletal Vertical** |  |  |
| SN-GoGn (°) | 0.979 | 0.988 |
| FMA (MP-FH) (°) | 0.981 | 0.976 |
| Mandibular Plane Angle (°) | 0.973 | 0.914 |
| Gonial Angle (°) | 0.971 | 0.936 |
| Ramus Height (mm) | 0.898 | 0.974 |
| **Dental Sagittal** |  |  |
| U1-SN (°) | 0.945 | 0.939 |
| U1-NA (°) | 0.976 | 0.921 |
| U1-NA (mm) | 0.965 | 0.971 |
| IMPA (°) | 0.963 | 0.947 |
| L1-NB (°) | 0.942 | 0.953 |
| L1-NB (mm) | 0.985 | 0.987 |
| Interincisal angle (°) | 0.908 | 0.935 |
| Overjet (mm) | 0.977 | 0.982 |
| U6 AP Position (mm) | 0.975 | 0.992 |
| L6 AP Position (mm) | 0.989 | 0.994 |
| U6 Angle (°) | 0.784 | 0.945 |
| U6-PTV (mm) | 0.971 | 0.945 |
| **Dental Vertical** |  |  |
| Overbite (mm) | 0.971 | 0.95 |
| **Soft Tissue** |  |  |
| Upper Lip to E-line (mm) | 0.983 | 0.979 |
| Lower Lip to E-line (mm) | 0.989 | 0.994 |
| Angle of Facial Convexity (°) | 0.986 | 0.986 |

**Supplementary Table 2.** Comparison of Herbst and Pendex at T2, Adjusted for Baseline (T1) Values of the Modeled Variable and Follow-Up Duration Between T1 and T2

| **Cephalometric Measure** | **Herbst** (n=23) | | **Pendex** (n=23) | | **Effect Size** | **p-value** |
| --- | --- | --- | --- | --- | --- | --- |
|  | **Mean** | **95% CI** | **Mean** | **95% CI** |  |  |
| ***Skeletal Sagittal*** | | | | | | |
| SNA (°) | 81.78 | 80.84,82.72 | 82.57 | 81.63,83.51 | -0.43 | 0.3067 |
| Midfacial Length (mm) | 84.44 | 83.18,85.69 | 85.51 | 84.26,86.76 | -0.46 | 0.3018 |
| SNB (°) | 78.33 | 77.42,79.24 | 78.06 | 77.15,78.97 | 0.15 | 0.7187 |
| Mandibular Length (mm) | 107.42 | 105.54,109.31 | 106.91 | 105.02,108.79 | 0.14 | 0.7375 |
| Mandibular Body Length (mm) | 65.79 | 64.49,67.10 | 65.11 | 63.80,66.41 | 0.27 | 0.5189 |
| Corpus Length (mm) | 69.54 | 68.24,70.84 | 69.22 | 67.92,70.53 | 0.12 | 0.7684 |
| ANB (°) | 3.72 | 3.14,4.30 | 4.45 | 3.87,5.03 | -0.69 | 0.1399 |
| Wits (mm) | -1.14 | -1.97,-0.30 | 1.82 | 0.99,2.66 | -1.94 | **0.0001** |
| Angle of Convexity (°) | 6.25 | 4.85,7.64 | 8.28 | 6.88,9.68 | -0.79 | 0.0865 |
| Mx/Md Difference (mm) | 23.30 | 22.03,24.57 | 21.08 | 19.81,22.35 | 0.90 | 0.0371 |
| ***Skeletal Vertical*** | | | | | | |
| SN-GoGn (°) | 31.55 | 30.36,32.74 | 30.53 | 29.34,31.73 | 0.44 | 0.2997 |
| FMA (MP-FH) (°) | 24.56 | 23.38,25.75 | 23.62 | 22.43,24.80 | 0.42 | 0.3364 |
| Mandibular Plane Angle (°) | 18.51 | 17.42,19.59 | 17.17 | 16.08,18.25 | 0.65 | 0.1381 |
| Gonial Angle (°) | 121.11 | 119.98,122.24 | 120.11 | 118.98,121.24 | 0.46 | 0.2789 |
| Ramus Height (mm) | 53.04 | 51.36,54.72 | 52.70 | 51.01,54.38 | 0.11 | 0.8045 |
| ***Dental Sagittal*** | | | | | | |
| U1-SN (°) | 103.33 | 100.43,106.24 | 102.11 | 99.21,105.02 | 0.22 | 0.6082 |
| U1-NA (°) | 21.65 | 19.01,24.29 | 19.42 | 16.78,22.06 | 0.44 | 0.3039 |
| U1-NA (mm) | 3.27 | 2.47,4.07 | 3.45 | 2.65,4.24 | -0.11 | 0.7896 |
| IMPA (°) | 95.24 | 92.51,97.97 | 96.50 | 93.77,99.23 | -0.25 | 0.5772 |
| L1-NB (°) | 27.02 | 24.47,29.58 | 27.84 | 25.28,30.39 | -0.17 | 0.7013 |
| L1-NB (mm) | 6.36 | 5.55,7.18 | 5.29 | 4.48,6.11 | 0.69 | 0.1180 |
| Interincisal Angle (°) | 127.28 | 122.58,131.97 | 128.62 | 123.93,133.32 | -0.16 | 0.7310 |
| Overjet (mm) | 2.20 | 1.60,2.80 | 4.18 | 3.58,4.78 | -1.93 | **0.0004** |
| U6 AP Position (mm) | 38.26 | 36.07,40.44 | 34.95 | 32.77,37.14 | 0.78 | 0.0688 |
| L6 AP Position (mm) | 40.74 | 38.99,42.49 | 37.82 | 36.07,39.57 | 0.86 | 0.0456 |
| U6 Angle (°) | 72.14 | 67.96,76.32 | 62.34 | 58.16,66.52 | 1.21 | 0.0059 |
| L6 Angle (°) | 81.30 | 78.60,84.00 | 81.66 | 78.96,84.37 | -0.07 | 0.8674 |
| U6-PTV (mm) | 16.38 | 15.30,17.47 | 15.27 | 14.19,16.36 | 0.53 | 0.2141 |
| ***Dental Vertical*** | | | | | | |
| Overbite (mm) | 1.12 | 0.38,1.85 | 2.93 | 2.20,3.67 | -1.27 | **0.0040** |
| U6 Vertical Position (mm) | -61.31 | -62.75,-59.87 | -61.66 | -63.10,-60.23 | 0.13 | 0.7643 |
| L6 Vertical Position (mm) | -62.56 | -64.03,-61.09 | -63.26 | -64.73,-61.79 | 0.25 | 0.5630 |
| ***Soft Tissue*** | | | | | | |
| Upper Lip to E-line (mm) | -3.31 | -4.30,-2.33 | -2.15 | -3.14,-1.17 | -0.62 | 0.1560 |
| Lower Lip to E-line (mm) | -0.19 | -1.17,0.79 | -0.27 | -1.25,0.71 | 0.04 | 0.9259 |
| Angle of Facial Convexity (g'-sn'-Pog) (°) | 153.18 | 151.90,154.46 | 149.42 | 148.14,150.71 | 1.58 | **0.0010** |
| **Bold** p-values were statistically significant at Hochberg’s adjusted α-level. All p-values reflect group differences from covariate-adjusted linear regression models. Effect size calculated as SMD = adjusted group difference ÷ model residual SD (RMSE); similar to Cohen’s d but based on regression-adjusted estimates (small: 0.2, medium: 0.5, large: 0.8). Effect size calculated as: Herbst – Pendex. | | | | | | |

**Supplementary Table 3.** Comparison of Herbst and Pendex Treatment Effects from T1 to T2, Adjusted for Baseline (T1) Values of the Modeled Variable and Follow-Up Duration Between T1 and T2

| **Cephalometric Measure** | **Herbst** (n=23) | | **Pendex** (n=23**)** | | **Effect Size** | **p-value** |
| --- | --- | --- | --- | --- | --- | --- |
|  | **Mean** | **95% CI** | **Mean** | **95% CI** |  |  |
| ***Skeletal Sagittal*** | | | | | | |
| SNA (°) | -1.29 | -2.01,-0.56 | 0.01 | -0.71,0.73 | -0.75 | 0.0141 |
| Midfacial Length (mm) | 2.76 | 1.31,4.20 | 2.69 | 1.24,4.13 | 0.02 | 0.9467 |
| SNB (°) | 0.64 | -0.21,1.49 | 1.17 | 0.33,2.02 | -0.27 | 0.3779 |
| Mandibular Length (mm) | 6.94 | 4.86,9.02 | 6.70 | 4.62,8.78 | 0.05 | 0.8701 |
| Mandibular Body Length (mm) | 3.62 | 2.44,4.80 | 4.75 | 3.57,5.93 | -0.41 | 0.1828 |
| Corpus Length (mm) | 3.29 | 2.19,4.39 | 4.38 | 3.28,5.48 | -0.42 | 0.1712 |
| ANB (°) | -1.63 | -2.22,-1.03 | -1.11 | -1.71,-0.52 | -0.38 | 0.2353 |
| Wits (mm) | -2.36 | -3.18,-1.53 | -0.73 | -1.55,0.09 | -0.90 | 0.0117 |
| Angle of Convexity (°) | -3.81 | -5.25,-2.36 | -3.24 | -4.68,-1.80 | -0.17 | 0.5863 |
| Mx/Md Difference (mm) | 4.15 | 2.96,5.33 | 4.05 | 2.86,5.23 | 0.04 | 0.9051 |
| ***Skeletal Vertical*** | | | | | | |
| SN-GoGn (°) | 0.29 | -0.92,1.50 | -1.15 | -2.36,0.06 | 0.50 | 0.0962 |
| FMA (MP-FH) (°) | -0.73 | -1.80,0.33 | -1.13 | -2.20,-0.07 | 0.16 | 0.5940 |
| Mandibular Plane Angle (°) | -0.56 | -1.63,0.52 | -1.28 | -2.36,-0.21 | 0.28 | 0.3422 |
| Gonial Angle (°) | -0.48 | -1.72,0.75 | 0.32 | -0.92,1.55 | -0.27 | 0.3638 |
| Ramus Height (mm) | 5.22 | 3.65,6.79 | 3.98 | 2.41,5.55 | 0.33 | 0.2682 |
| ***Dental Sagittal*** | | | | | | |
| U1-SN (°) | 3.29 | 0.78,5.81 | 5.38 | 2.86,7.89 | -0.36 | 0.2626 |
| U1-NA (°) | 4.71 | 2.12,7.30 | 5.19 | 2.60,7.78 | -0.08 | 0.8021 |
| U1-NA (mm) | 0.61 | -0.33,1.55 | 1.07 | 0.13,2.01 | -0.22 | 0.5131 |
| IMPA (°) | 0.52 | -2.19,3.24 | 0.69 | -2.02,3.41 | -0.03 | 0.9326 |
| L1-NB (°) | 0.73 | -1.54,3.00 | 1.45 | -0.82,3.73 | -0.14 | 0.6595 |
| L1-NB (mm) | 1.34 | 0.64,2.04 | 0.90 | 0.20,1.60 | 0.27 | 0.3773 |
| Interincisal Angle (°) | -4.40 | -8.12,-0.69 | -4.91 | -8.62,-1.20 | 0.06 | 0.8576 |
| Overjet (mm) | -1.88 | -2.43,-1.34 | -1.98 | -2.53,-1.44 | 0.10 | 0.8261 |
| U6 AP Position (mm) | 2.35 | 0.71,4.00 | 3.23 | 1.59,4.88 | -0.22 | 0.4513 |
| L6 AP Position (mm) | 4.10 | 2.42,5.78 | 3.86 | 2.18,5.54 | 0.06 | 0.8396 |
| U6 Angle (°) | 1.78 | 0.06,3.51 | 4.14 | 2.41,5.87 | -0.57 | 0.0581 |
| L6 Angle (°) | -4.15 | -6.32,-1.99 | -5.33 | -7.49,-3.17 | 0.23 | 0.4443 |
| U6-PTV (mm) | 3.59 | 2.45,4.73 | 4.65 | 3.51,5.79 | -0.39 | 0.1921 |
| ***Dental Vertical*** | | | | | | |
| Overbite (mm) | -1.61 | -1.98,-1.24 | -2.06 | -2.43,-1.70 | 0.52 | 0.0834 |
| U6 Vertical Position (mm) | -4.60 | -5.93,-3.26 | -4.30 | -5.64,-2.97 | -0.09 | 0.7544 |
| L6 Vertical Position (mm) | -5.07 | -6.41,-3.74 | -4.67 | -6.01,-3.34 | -0.13 | 0.6729 |
| ***Soft Tissue*** | | | | | | |
| Upper Lip to E-line (mm) | -2.22 | -2.99,-1.45 | -2.12 | -2.89,-1.35 | -0.06 | 0.8557 |
| Lower Lip to E-line (mm) | -0.54 | -1.40,0.32 | -0.95 | -1.81,-0.09 | 0.21 | 0.5045 |
| Angle of Facial Convexity  (g'-sn'-Pog) (°) | 1.32 | 0.08,2.55 | 0.86 | -0.38,2.09 | 0.16 | 0.6078 |
| No p-values were statistically significant at Hochberg’s adjusted α-level. All p-values reflect group differences from covariate-adjusted linear regression models. Effect size calculated as SMD = adjusted group difference ÷ model residual SD (RMSE); similar to Cohen’s d but based on regression-adjusted estimates (small: 0.2, medium: 0.5, large: 0.8). Effect size calculated as: (T3−T1 change in Herbst) − (T3−T1 change in Pendex). | | | | | | |
